# Large set data mining reveals overexpressed GPCRs in prostate and breast cancer: potential for active targeting with engineered anti-cancer nanomedicines

**DOI:** 10.18632/oncotarget.25427

**Published:** 2018-05-18

**Authors:** Eric Kübler, Hugo Albrecht

**Affiliations:** ^1^ Institute for Chemistry and Bioanalytics, School of Life Sciences, University of Applied Sciences and Arts Northwestern Switzerland, Muttenz 4132, Switzerland; ^2^ Centre for Pharmaceutical Innovation and Development, Centre for Drug Discovery and Development, Sansom Institute for Health Research, School of Pharmacy and Medical Sciences, University of South Australia, Adelaide, SA 5001, Australia

**Keywords:** GPCR, cancer, nanocarrier, meta-data, targeted chemotherapy, Pathology

## Abstract

Over 800 G-protein-coupled receptors (GPCRs) are encoded by the human genome and many are overexpressed in tumors. GPCRs are triggered by ligand molecules outside the cell and activate internal signal transduction pathways driving cellular responses. The receptor signals are desensitized by receptor internalization and this mechanism can be exploited for the specific delivery of ligand-linked drug molecules directly into cells. Detailed expression analysis in cancer tissue can inform the design of GPCR-ligand decorated drug carriers for active tumor cell targeting. The active targeting process utilizes ligand receptor interactions leading to binding and in most cases internalization of the ligand-attached drug carrier resulting in effective targeting of cancer cells. In this report public microarray data from the Gene Expression Omnibus (GEO) repository was used to identify overexpressed GPCRs in prostate and breast cancer tissues. The analyzed data confirmed previously known cancer receptor associations and identified novel candidates for potential active targeting. Prioritization of the identified targeting receptors is also presented based on high expression levels and frequencies in cancer samples but low expression in healthy tissue. Finally, some selected examples were used in ligand docking studies to assess the feasibility for chemical conjugation to drug nanocarriers without interference of receptor binding and activation. The presented data demonstrate a large untapped potential to improve efficacy and safety of current and future anti-cancer compounds through active targeting of GPCRs on cancer cells.

## INTRODUCTION

Globally, more than 14 million cancer-related deaths were reported in 2012 and this annual burden is expected to grow to approximately 22 million by 2030 (World Cancer Report 2014, Stewart BW, Wild CW, ISBN 978-92-832-0443-5). Currently available anti-cancer drugs are inadequate, and there is demand for better formulations to reduce undesirable adverse effects. The major problem with traditional cytotoxic drugs is their poor bio-distribution to tumors leading to toxic side effects against healthy cells. A consequence of this is that the optimal dose often cannot be administered. Additional challenges are posed by low solubility, rapid *in vivo* breakdown of free drug, and tissue damage on extravasation. Even molecular targeted cancer therapies (e.g. kinase inhibitors) show side effects and lack of selectivity for neoplastic tissue. Potentially, most of these issues can be overcome by delivery of anti-cancer agents with engineered nanoparticles targeting surface proteins which are overexpressed on cancer cells, but show much lower or no expression in healthy tissue [1]. The majority of clinically available anti-cancer nano-formulations use passive targeting, exploiting the Enhanced Permeability and Retention Effect (EPR) [2]. In this case, passive diffusion through endothelial fenestrations of tumor tissue lead to a local build-up of nanoparticle concentrations, an effect further enhanced by the lack of efficient lymphatic drainage. However, nanoparticles also accumulate in various organs, mainly liver and spleen, by vascular escape through endothelial fenestrations [3]. To minimize this effect, drug carriers can be functionalized with ligands or antibodies for active targeting of receptors which show overexpression on cancer cells in comparison to healthy tissue. This can both further enhance the anti-cancer potency on solid tumors and reduce toxic side effects on healthy cells.

Tumor cells generally show a characteristic pattern of overexpressed membrane associated proteins such as receptors, membrane transporters and adhesion molecules. G-protein-coupled-receptors are the largest family of trans-membrane receptors and some are known to be overexpressed in prevalent solid tumors. The most intensely studied targeting receptors from the GPCR family are the somatostatin [4–6], cholecystokinin [7, 8], gastrin-releasing peptide (GRP) [9–11], lutein releasing hormone [12, 13], and neurotensin receptors [14, 15]. Considering the number of known GPCR receptor family members, they appear to be under-represented in current research addressing active receptor targeting. We believe that many more GPCR ligands could be exploited to design drug carriers that e.g. trigger receptor internalization and hence nanoparticle and anti-cancer agent delivery directly into endosomal compartments.

**Figure 1:**
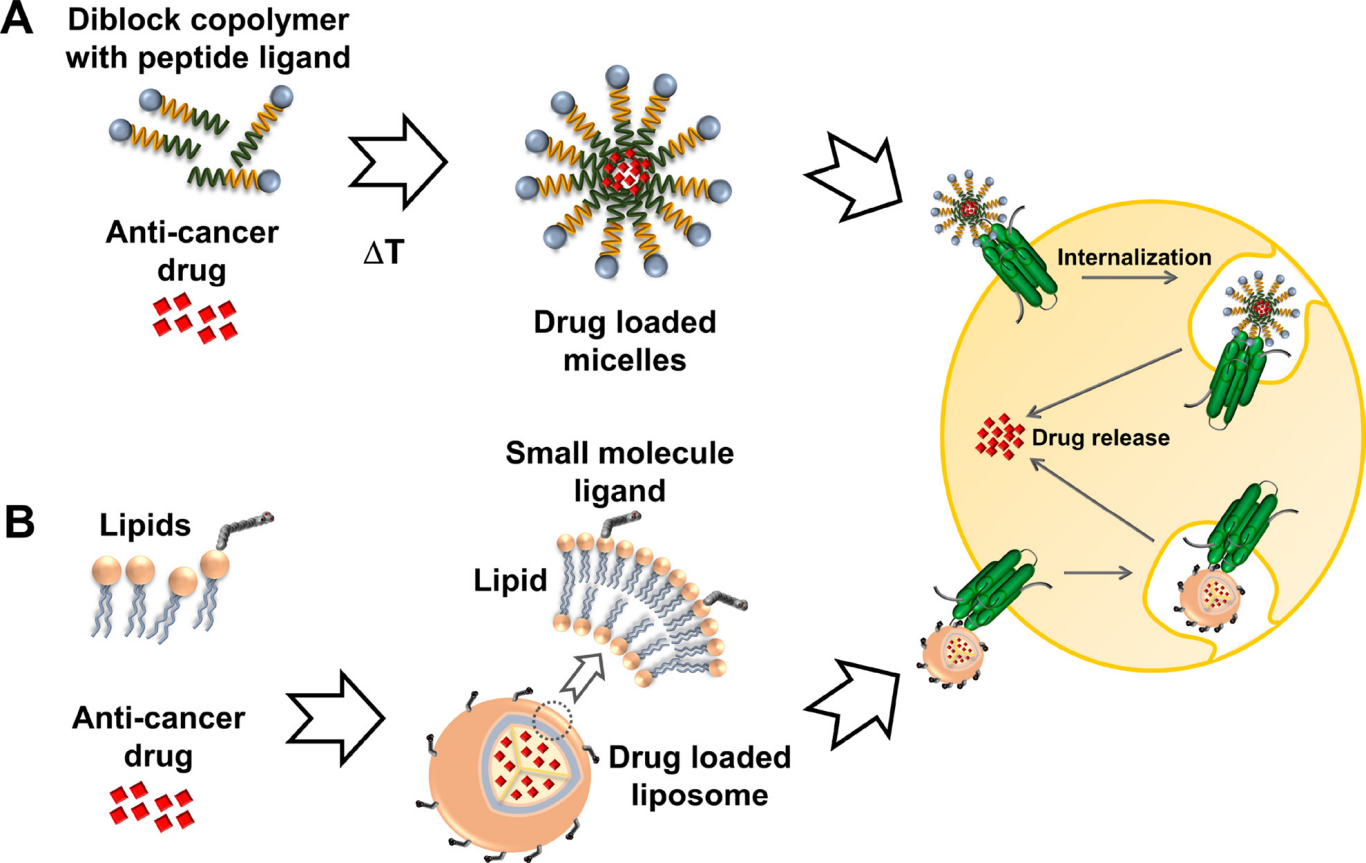
Active targeting of cancer cells Nanoparticles are decorated with ligands for specific docking to GPCRs overexpressed in cancer cells and driving cellular uptake via receptor internalization. (**A**) ELPs are self-assembling diblock copolymers which can be engineered as fusion proteins with a tethered peptide ligand (depicted as blue sphere). (**B**) Small molecule ligands can be covalently attached to lipid components such as phosphatidylcholine for display on the surface of drug loaded liposomes.

In the past most strategies for drug carrier development focused on liposomal, polymeric and inorganic formulations. Doxil/Caelyx was the first FDA approved anti-cancer nanomedicine [16] and in the meantime is one of seven clinically approved liposomal cancer treatments [17]. In contrast, polymer-based formulations have lagged behind, with Abraxane [18] and Genexol-PM [19] the first FDA approved examples in 2005 and 2007, respectively. Efforts to translate various polymer formulations from preclinical setting to clinical application are ongoing [1]. Notably, the use of amphiphilic synthetic and natural diblock copolymers, which self-assemble into nanoparticles has been recently reported [20]. Elastin-like polypeptides (ELP) are an example of natural diblock copolymers that can be genetically engineered with attached peptide ligands, offering a strategy amenable to >150 GPCRs (Figure 1A) [21–23]. Alternatively, ligands can also be chemically conjugated with methods analogous to those commonly used with liposomes (Figure 1B).

Here we present the identification of GPCRs overexpressed in primary prostate and breast cancer tissue. The suitability of overexpressed receptors for specific cancer cell targeting with drug nanocarriers was assessed by consideration of the expression levels in cancer vs healthy tissue. Structural insight is given for selected receptors to indicate amenability for chemical conjugation to nanoparticles. Our findings reveal a large untapped potential for GPCR targeting in cancer treatment with engineered drug carriers.

## RESULTS

### Collection of gene expression data

**Figure 2:**
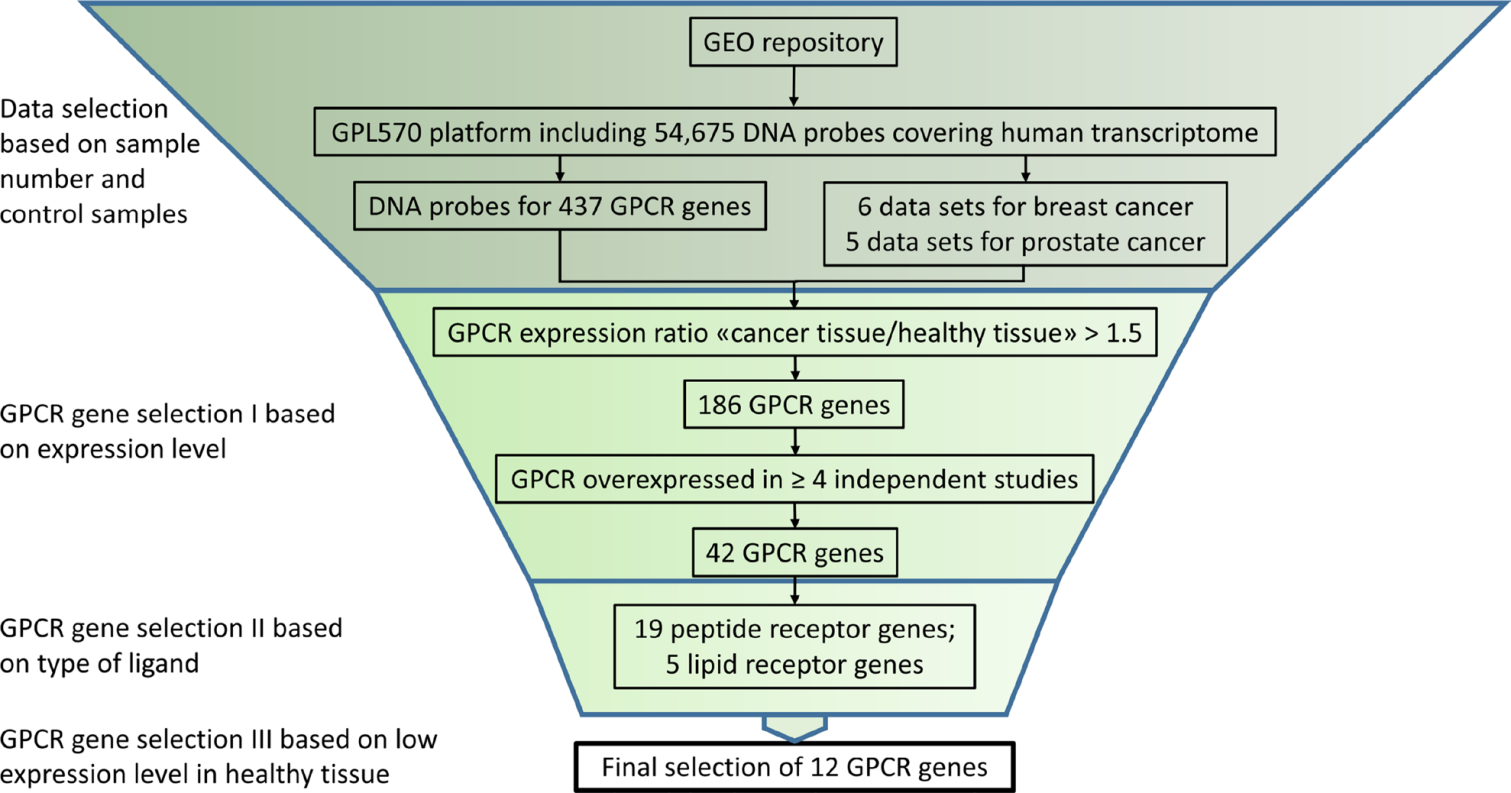
*In silico GPCR* gene selection algorithm Suitable GLP570 based data sets were selected and a diverse set of inclusion criteria were applied to select a final set of 12 GPCR genes.

The aim of this study was to analyze prostate and breast cancer tissue for the overexpression of GPCR receptors with the goal of identifying potential targets for active delivery of anti-cancer compounds with GPCR-ligand guided drug carriers. The public GEO repository was systematically searched with a focus on a single DNA microarray platform to obtain easily compared data. For this purpose, the GPL570 human DNA array (Affymetrix Inc., Santa Clara, CA, USA) containing 54,675 DNA probes covering most of the human transcriptome was selected. The chosen platform represented >120,000 samples available from the GEO data base. A list of 755 GPCR genes was compiled from 21 Pfam domains (7tm and GPCR) within the protein family database (http://pfam.sanger.ac.uk/) and 437 GPCR genes were identified on the GPL570 platform. The 318 genes not found were largely accounted for by olfactory receptors which were not represented on the GPL570 platform. All over, the chosen platform contained >90% of all non-olfactory GPCRs, which was considered suitable for the study. An initial search for prostate and breast cancer data sets in the GEO repository limited to the GPL570 platform resulted in 28 and 37 hits, respectively. The data sets were checked for suitability according to the criteria outlined in the methods section and non-informative data sets have been excluded. The final selection of 6 prostate and 5 breast cancer data sets is given in Table 1. Figure 2 shows the selection cascade outlining the applied criteria which lead to the final selection of overexpressed receptors.

**Table 1:** Gene expression datasets

Data set accession	Sample size	Primary cancer samples
Prostate cancer		
GDS1439	19	Benign prostate (6); clinically localized primary (7) and metastatic prostate cancer (6) [43]
GDS4114	12	Normal prostate stroma (6); Stroma associated to prostate invasive tumor (6) [44]
GDS4824	21	Normal benign prostate (8); malignant TMPRSS2:ERG fusion negative (7) and TMPRSS2:ERG fusion positive (6) [45]
GSE17951	126	Control biopsy (17); Radical prostatectomy with 0% (41), <50% (38) and >50% tumor cells (30) [46]
GSE32448	80	Normal well differentiated (20) and poorly differentiated (20)^1)^; tumor well differentiated (20) and poorly differentiated (20) [47]
GSE46602	50	Tissue from benign prostate glands (10); benign adjacent to prostate cancer (4); prostate cancer tissue (36) [48]
Breast cancer		
GSE8977	22	Stroma normal breast (15); stroma tumor (7) [49]
GSE22544	20	Normal breast tissue (4); IDC (14); node metastasis (2) [50]
GSE29431	66	Normal breast tissue (12); primary breast carcinomas (54)^2)^ [51]
GSE42568	121	Normal breast tissue (17); [IDC (82); invasive lobular (17); tubular (2); mucinous (3)]^3)^ [52]
GSE61304	62	Normal breast epithelium (4); breast tumor epithelium (52) [53]

IDC: infiltrating ductal carcinoma; ^1)^poorly differentiated samples were excluded; ^2)^these 54 samples were split into 3 groups according to Her2 IHC scores (0/1, 2, 3); ^3)^Pooled into 1 group of 104 samples, calculated vs 17 normal samples (1 calculation only for GSE42568); the number of samples are indicated in brackets.

### Identification of overexpressed GPCRs

**Figure 3:**
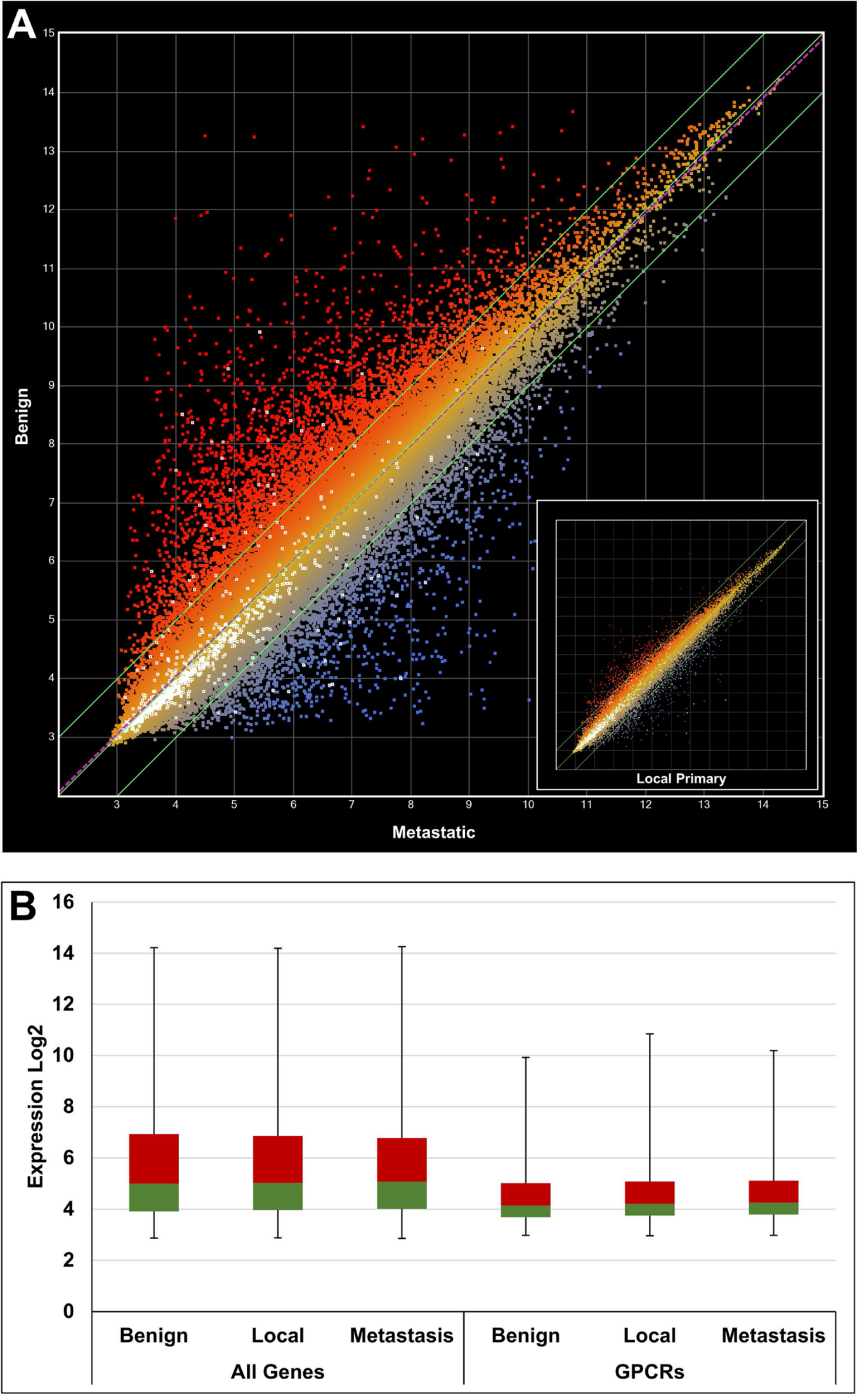
Gene expression analysis using a scatter plot to compare benign, local and metastatic tumor samples from the GDS1439 data set (**A**) Benign vs metastatic (*R*^2^ = 0.8734) and inset showing benign vs primary local (*R*^2^ = 0.9760). White dots indicate GPCR genes. (**B**) Box plot comparing expression of all genes vs GPCRs only. The 2nd and 3rd quartiles are colored in green and red, respectively. The first and fourth quartiles are indicated by error bars. A general lower expression level of GPCR genes can be deduced.

**Figure 4:**
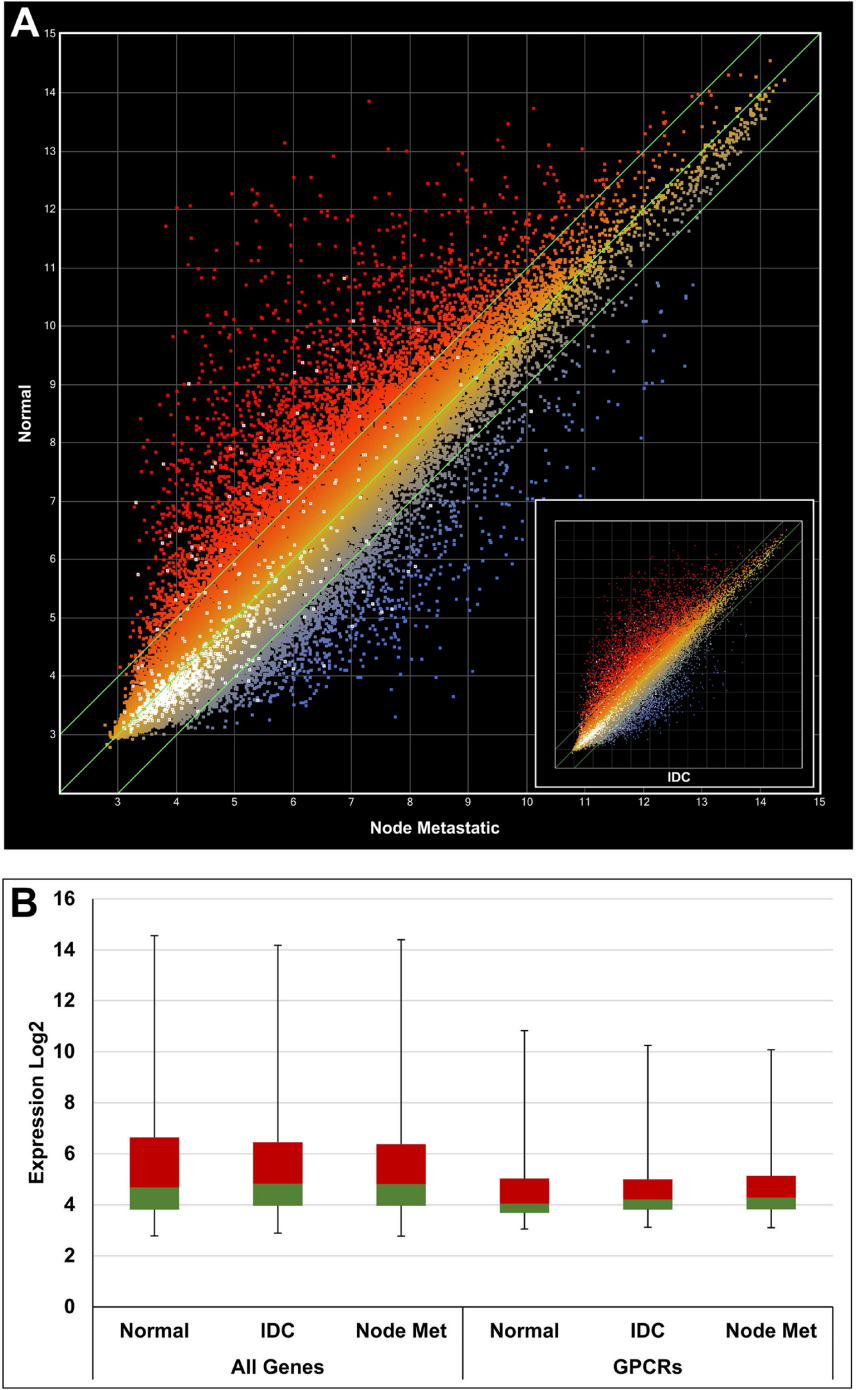
Gene expression analysis using a scatter plot with data from GSE22544 (**A**) Normal vs node metastasis *R*^2^ = 0.8663. Inset: Normal vs IDC *R*^2^ = 0.8657. White dots indicate GPCR genes. (**B**) Box plot comparing expression of all genes vs GPCRs. The 2nd and 3rd quartiles are colored in green and red, respectively. The first and fourth quartiles are indicated by error bars. A general lower expression level of GPCR genes can be deduced. IDC: infiltrating ductal carcinoma.

CEL data files were retrieved from the public GEO repository and processed using a robust multi-array (RMA) [24] normalization protocol with the ArrayStar software (DNAStar Inc, Madison, WI, USA). Annotations and attributes were imported automatically from files provided by Affymetrix Inc. (Santa Clara, CA, USA). Overexpressed GPCRs were systematically identified by comparison of samples from cancer and healthy tissues, e.g. metastatic prostate cancer vs benign prostate or breast node metastasis vs normal breast tissue and so forth. For each data set one or more calculations have been performed. A total of 12 calculations were carried out for prostate cancer samples and 8 calculations for breast cancer samples. A representative example (GDS1439) for prostate cancer is illustrated with the scatter plot in Figure 3A. In this case 2 calculations were performed, benign vs metastatic tissue and benign vs local primary tissue. The sample numbers for benign, localized primary and metastatic tissue were 6, 7 and 6, respectively, as indicated in brackets of Table 1. The color coding indicates overexpression in metastatic prostate cancer samples (blue) and overexpression in benign prostate cancer cells (red). All GPCR probes are highlighted as individual white dots. The differential expression was stronger in metastatic prostate cancer samples (*R*^2^ = 0.8734) in comparison to local primary prostate cancer samples (*R*^2^ = 0.9760) as shown in the inset of Figure 3A. An additional example is given for breast cancer in Figure 4A, and in this case the differential expression was similar when normal tissue was compared to node metastasis and invasive ductal carcinoma (IDC) as indicated by very similar *R*^2^ values of 0.8663 and 0.8657, respectively.

It was apparent from the scatter plots that the average GPCR expression levels were lower than the total average expression. This has been confirmed by different quartile distributions as shown in the box plots of Figures 3B and 4B.

Overall, 186 GPCR genes were identified with ≥1.5 fold expression in cancer compared to healthy control tissues in at least one calculation. At this stage, genes with more than one corresponding DNA probe on the GPL570 platform but only a single one rendering a positive signal were included. It was assumed that DNA probes with positive signals in multiple cancer tissues will be more significant. Therefore, as additional criteria, only genes with overexpression in at least 4 replica groups of cancer tissue were considered for further analysis. This arbitrary cut-off was applied to discount all genes with overexpression in less than 4 cancer tissue groups, resulting in a reduction of the initial gene set to a total of 42 overexpressed genes, 11 in PCa (Table 2), 24 in BrCa (Table 3) and 7 genes in both PCa and BrCa. The gene symbols, probe IDs and fold increase are summarized in Tables 2 and 3.

**Table 2:** Overexpression of G-proteins in prostate cancer tissue (min 4 × >1.5 fold induction)

Gene	Probe ID	Fold increase (*p*-Value)
ADGRV1^a)^	223582_at	1.81 (0.024), 1.59 (0.222), 1.73 (0.016), 1.95 (0.016)
	234871_at	1.67 (0.958)
ADRA2A	209869_at	3.39 (0.074), 1.54 (0.539), 1.84 (0.002), 1.81 (0.017), 1.80 (0.017)
ADRB1	229309_at	3.59 (0.034), 1.81 (0.387), 1.91 (0.355), 2.07 (0.411), 2.65 (0.162), 2.57 (0.000), 1.75 (0.000)
	229277_at	1.79 (0.339), 1.64 (0.425)
AGTR1	205357_s_at	1.70 (0.481), 1.54 (0.108), 2.51 (0.000), 1.75 (0.001)
	208016_s_at	1.50 (0.112), 1.77 (0.039), 2.04 (0.272)
C3AR1	209906_at	1.55 (0.627), 1.80 (0.000), 2.03 (0.000), 1.77 (0.000)
CELSR3^a)^	40020_at	5.09 (0.011), 1.56 (0.408), 1.53 (0.485), 1.59 (0.034)
	205165_at	4.72 (0.012)
CHRM3	1553705_a_at	3.04 (0.027), 1.53 (0.061), 1.83 (0.260), 4.26 (0.045), 1.59 (0.048), 2.67 (0.000), 2.08 (0.077), 1.81 (0.000), 1.68 (0.000)
	214596_at	4.89 (0.018), 1.76 (0.113), 1.97 (0.407), 6.75 (0.037), 1.94 (0.036), 3.51 (0.000), 3.30 (0.020), 3.42 (0.002), 3.96 (0.002)
	242488_at	6.32 (0.020), 1.69 (0.176), 1.71 (0.593), 8.25 (0.038), 2.13 (0.039), 5.21 (0.002), 3.16 (0.002), 3.27 (0.002)
	1559633_a_at	4.11 (0.047), 1.52 (0.650), 4.45 (0.135), 2.08 (0.003), 2.89 (0.000), 2.53 (0.000)
	1564339_a_at	2.71 (0.023), 1.83 (0.000), 1.72 (0.000)
	1559634_at	1.66 (0.128), 1.67 (0.000), 1.57 (0.007)
CXCR4^a)^	211919_s_at	2.45 (0.220), 6.63 (0.000), 8.52 (0.000), 6.06 (0.000)
	209201_x_at	2.56 (0.194), 6.46 (0.000), 8.34 (0.000), 6.06 (0.000)
	217028_at	2.95 (0.195), 2.53 (0.000), 2.47 (0.000), 2.29 (0.000)
F2R	203989_x_at	1.55 (0.150), 1.58 (0.023), 1.54 (0.667), 1.60 (0.145), 1.87 (0.000), 1.66 (0.002), 1.76 (0.002)
F2RL1^a)^	213506_at	3.30 (0.039), 2.02 (0.149), 2.95 (0.507), 1.90 (0.001), 1.90 (0.000)
	206429_at	1.88 (0.024), 2.20 (0.000), 2.10 (0.000)
F2RL2^a)^	230147_at	2.58 (0.653), 2.21 (0.166), 1.98 (0.304), 1.67 (0.013), 2.30 (0.000)
FPR1	205119_s_at	1.64 (0.002), 3.22 (0.000), 3.31 (0.000), 2.10 (0.000)
FPR3^a)^	214560_at	1.67 (0.002), 1.54 (0.292)
	230422_at	2.44 (0.340), 2.05 (0.000), 2.33 (0.000), 2.16 (0.000)
GPR183	205419_at	1.52 (0.351), 3.41 (0.000), 3.65 (0.000), 2.42 (0.000)
GRPR^a)^	207929_at	1.89 (0.189), 1.60 (0.002), 2.18 (0.000), 1.69 (0.006)
OR51E1	229768_at	13.30 (0.012), 2.64 (0.441), 3.22 (0.259), 2.19 (0.535), 2.82 (0.842)
OR51E2	221424_s_at	14.38 (0.003), 3.96 (0.037), 4.66 (0.321), 4.55 (0.284), 5.44 (0.000), 6.08 (0.000), 1.51 (0.203), 4.86 (0.006), 2.33 (0.006)
	232482_at	29.45 (0.001), 6.80 (0.072), 2.38 (0.178), 4.18 (0.290), 3.92 (0.271), 4.33 (0.000), 4.92 (0.000), 1.88 (0.279), 3.38 (0.094), 4.15 (0.001), 2.57 (0.001)
	236121_at	36.83 (0.003), 6.10 (0.236), 24.45 (0.171), 2.98 (0.406), 3.50 (0.329), 2.28 (0.006), 2.43 (0.001), 5.04 (0.207), 8.96 (0.083), 3.57 (0.022), 1.74 (0.022)
SSTR1	235591_at	2.18 (0.325), 2.32 (0.150), 2.54 (0.000), 1.83 (0.071)
	208482_at	1.75 (0.000)

^a)^Overexpressed in PCa and BrCa.

**Table 3:** Overexpression of G-proteins in breast cancer tissue (min 4 × >1.5 fold induction)

Gene	Probe ID	Fold increase (*p*-Value)
ADGRV1	223582_at	2.02 (0.063), 2.04 (0.297), 2.29 (0.021), 1.59 (0.106), 1.53 (0.276), 1.62 (0.000)
	224275_at	1.55 (0.024)
ADORA3	206171_at	1.89 (0.062), 2.20 (0.010), 2.75 (0.000), 2.23 (0.032)
CCR1	205098_at	1.97 (0.090), 1.92 (0.105), 1.61 (0.136), 1.68 (0.265)
	205099_s_at	1.80 (0.026)
CCR2	206978_at	1.56 (0.228), 1.65 (0.729), 1.65 (0.181), 2.03 (0.051)
CCR5	206991_s_at	1.74 (0.011), 2.69 (0.359), 2.80 (0.001), 2.08 (0.003), 1.90 (0.030), 1.69 (0.000), 1.88 (0.002)
CCR7	206337_at	2.80 (0.242), 3.68 (0.638), 1.86 (0.249), 1.58 (0.001), 2.52 (0.024)
CELSR1	41660_at	5.79 (0.003), 3.37 (0.122), 2.56 (0.014), 4.21 (0.000), 4.51 (0.001), 5.93 (0.000)
CELSR2	36499_at	6.02 (0.000), 5.31 (0.047), 1.80 (0.130), 2.48 (0.080), 2.78 (0.000)
	204029_at	4.15 (0.001), 4.33 (0.076), 1.68 (0.135), 2.51 (0.066), 2.14 (0.000)
CELSR3	40020_at	2.36 (0.006), 1.54 (0.071), 1.98 (0.000), 1.55 (0.085), 1.73 (0.000), 1.81 (0.000)
CXCR3	207681_at	1.94 (0.548), 1.66 (0.024), 1.56 (0.025), 1.63 (0.000)
CXCR4	211919_s_at	2.67 (0.017), 2.68 (0.061), 4.60 (0.525), 2.14 (0.047), 3.29 (0.000), 2.45 (0.006), 1.60 (0.002), 2.96 (0.000)
	209201_x_at	2.66 (0.016), 2.54 (0.087), 4.54 (0.480), 2.69 (0.013), 3.85 (0.000), 2.94 (0.002), 1.62 (0.002), 2.62 (0.000)
	217028_at	2.51 (0.036), 3.18 (0.004), 2.89 (0.664), 2.47 (0.011), 2.27 (0.006), 2.10 (0.043), 2.79 (0.000), 1.83 (0.032)
F2RL1	213506_at	2.06 (0.267), 1.81 (0.643), 2.52 (0.033), 3.31 (0.000), 3.92 (0.009), 2.42 (0.000)
F2RL2	230147_at	1.54 (0.573), 4.43 (0.662), 3.60 (0.048), 3.87 (0.008), 8.53 (0.000), 3.22 (0.000)
FPR3	214560_at	2.05 (0.043), 1.69 (0.002), 2.22 (0.000), 1.80 (0.032), 2.17 (0.000)
	230422_at	1.77 (0.259), 1.63 (0.194), 1.70 (0.260), 3.58 (0.002)
GPR171	207651_at	2.23 (0.245), 5.04 (0.503), 1.91 (0.220), 1.59 (0.402)
GPR18	210279_at	2.51 (0.405), 1.60 (0.066), 1.89 (0.052), 1.95 (0.006)
GPR19	207183_at	1.68 (0.012), 1.56 (0.057), 1.58 (0.003), 1.72 (0.023), 2.28 (0.000)
GPR37	209631_s_at	2.77 (0.022), 1.96 (0.097), 2.29 (0.034), 1.71 (0.000)
GPR39	229105_at	1.70 (0.135), 3.35 (0.011), 1.86 (0.081), 2.04 (0.152), 2.67 (0.000)
	229104_s_at	1.73 (0.030)
GPR65	214467_at	1.95 (0.109), 1.60 (0.032), 1.76 (0.041), 1.55 (0.014)
GPR68	229055_at	2.31 (0.009), 1.79 (0.031), 1.79 (0.241), 2.20 (0.000), 2.13 (0.000), 1.90 (0.012), 1.82 (0.000), 1.63 (0.000)
GPR84	223767_at	1.80 (0.102), 1.55 (0.015), 1.70 (0.057), 1.70 (0.000), 1.90 (0.015), 2.66 (0.000)
GPRC5A	203108_at	2.33 (0.175), 3.90 (0.013), 5.26 (0.000), 4.35 (0.015), 5.52 (0.000), 4.31 (0.000)
	212444_at	4.09 (0.005), 8.21 (0.000), 3.73 (0.019), 3.38 (0.000), 2.13 (0.013)
GRPR	207929_at	1.78 (0.003), 1.66 (0.143), 1.66 (0.001), 1.61 (0.035)
KISS1R	242517_at	2.47 (0.103), 1.82 (0.196), 4.46 (0.039), 2.58 (0.000), 1.65 (0.181)
LPAR2	206723_s_at	2.48 (0.008), 1.72 (0.199), 1.67 (0.184), 1.55 (0.086), 2.28 (0.000)
LPAR5	230252_at	2.00 (0.565), 1.82 (0.003), 1.79 (0.000), 1.90 (0.001)
OPN3	224392_s_at	2.77 (0.006), 1.92 (0.037), 2.16 (0.041), 1.55 (0.000)
	219032_x_at	1.20 (0.212), 1.68 (0.059), 1.95 (0.151), 1.74 (0.000), 1.55 (0.000)
P2RY2	206277_at	1.82 (0.030), 1.56 (0.025), 1.91 (0.000), 1.80 (0.004), 1.68 (0.005)
P2RY10	236280_at	1.57 (0.396), 3.49 (0.563), 3.50 (0.006), 2.83 (0.003), 2.71 (0.012), 1.59 (0.005), 1.77 (0.021)
	1553856_s_at	1.94 (0.031), 1.66 (0.036), 1.68 (0.000)
	214615_at	1.55 (0.064), 1.58 (0.005), 1.55 (0.000)
S1PR3	228176_at	2.20 (0.016), 2.42 (0.000), 2.02 (0.042), 1.72 (0.007)

Peptide receptors dominated the selected gene set (19 out of 42). This receptor class represents a high potential for active targeting as they bind relatively large ligands with ample opportunity for chemical linking to carriers. Five lipid receptors were identified, interestingly in BrCa only, and this type of ligand harbors significant potential for conjugation to nanoparticle based drug carriers. A total of 6 small molecule receptors were identified, namely ADORA3, P2RY2 and P2RY10 in BrCa; ADRA2A, ADRB1 and CHRM3 in PCa. It is likely that small ligands would represent a challenge to link with nanoparticles while maintaining activity on the receptor. Therefore we did not follow this group further. Two olfactory receptors were identified in PCa with OR51E2 very strongly and frequently expressed. However, this type of receptor is unlikely to be useful for the active targeting of nanoparticles due to the fact that in most cases the detected ligands are not known or are small molecules. In this case targeting may be considered with specific antibodies. However, this is beyond the scope of the current report and is not further discussed. Orphan receptors can be of future interest once the native ligands have been discovered. Our investigations revealed one adhesion (ADGRV1) and three cadherin type (CelsR1-3) orphan GPCRs (two in PCa and BrCa, and two in BrCa only). Of particular interest are the CelsR1-3 receptors which have shown very frequent and strong expression. In addition to this the orphan receptor GPRC5A showed very high and frequent expression in BrCa and GPR183 was moderately expressed in some PCa tissue. The future potential of orphan receptors is exemplified in this study by several recently de-orphaned members which have been identified as part of this project (e.g. GPR18, 19, 37 and 171) [25–28]. GPR65 is an additional recently de-orphaned, pH sensitive receptor and was deemed as too challenging for chemical conjugation purposes [29]. However, in the following sections the focus is on peptide and lipid receptors as these types show the highest potential for active targeting purposes in the near future. A summary of the identified receptors with peptide and lipid ligands is shown in Table 4.

**Table 4:** Final selection of receptors for future targeting of cancer cells

Receptor	Ligand	Current role in cancer treatment
Prostate cancer		
AGTR1	Angiotensin II	Anti angiogenic, PCa clinical pilot study [54–56]
F2R	Thrombin activated	None
FPR1	N-formyl peptides	None
Breast cancer		
CCR7	CCL19, 21	None
CXCR3	CXCL9, 10, 11	None
GPR18	N-arachidonyl glycine	None, recently de-orphaned
GPR19	Adropin	None, recently de-orphaned
GPR37	Prosaptide	None, recently de-orphaned
GPR171	BigLEN	None, recently de-orphaned
KISS1R	Kisspeptin	None
Prostate and breast cancer		
F2RL2	Thrombin activated	None
GRPR	GRP	PCa and others, diagnosis, radio and chemotherapy [54, 57]

### GPCRs with peptide ligands

Of all overexpressed GPCRs, 45% were peptidic receptors, five specific to PCa, nine specific to BrCa and five overexpressed in PCa and BrCa. The latter group contained the GRP receptor previously reported to be frequently overexpressed in prostate and breast cancer tissue [30]. Our data selection algorithm also identified the somatostatin receptor SSTR1 in PCa tissue. A peptidic somatostatin receptor ligand (octreotide) has been reportedly coupled with lutetium-177 and tested in phase 2 trials for the radio treatment of neuroendocrine tumors [31]. These results clearly validate the presented approach. The next step was to select the most promising receptors for effective and safe cancer cell targeting. This is most likely achieved using receptors with low expression in healthy tissue and high expression in neoplastic tissue. The information for receptor expression in healthy tissues was collected from the Human Protein Atlas (HPA) and is shown in Supplementary Data 1 with RNAseq data given for each receptor in the right column, and protein levels are indicated in the left hand column for each receptor, with 0 (not detected), 1 (low), 2 (medium) and 3 (high protein expression). From all selected receptors which were identified as overexpressed in PCa cells, we judged AGTR1 as most favorable as expression in healthy individuals was detected in only three tissue types, followed by FPR1 and F2R which showed expression in 15 out of 45 tissues. FPR1 showed expression at low levels only, with the exception of bone marrow. Similarly, the same kind of analysis of receptors with overexpression in BrCa cells revealed CCR7, CXCR3, GPR171 and GPR37 to be of most interest as they were only expressed in very few tissues of healthy individuals. A total of five receptors were identified in PCa & BrCa and protein expression data were only available for F2RL1 which was deemed unsuitable due to ubiquitous expression in most tissues. It has been well documented that protein and mRNA expression do not always correlate. Nevertheless, in the absence of information for protein expression we assessed mRNA expression and noted very limited expression of GRPR and KISS1R in almost all tissues. These receptors might be most suitable for selective and active targeting of cancer cells. To a lesser extent GPR19 and F2RL2 might also be considered.

### Bioactive lipid receptors

Interestingly, overexpressed lipid receptors were only detected in breast cancer tissue. The recently de-orphaned GPR18 showed medium protein expression in spleen, lymph node and tonsil (Supplementary Data 1). This was further confirmed by RNAseq which was limited to immune tissue (Supplementary Data 1). All other lipid receptors showed ubiquitous expression in most tissues and were therefore considered potentially problematic for selective targeting of cancer cells.

Many GPCRs have been reported to affect cancer relevant mechanisms. Surprisingly, only very few drugs have been launched to modulate these receptors for the purpose of cancer treatment [32], and to date even fewer receptors have been used for active targeting. A summary of all receptors selected from this study as potential cancer cell targeting options are summarized in Table 4.

### Ligand docking

A ligand can only guide drug carriers to target cells if the bound ligand protrudes from the receptor in a way that displays functional groups amenable for chemical conjugation. Docking experiments were conducted with selected ligands to gain structural insight into the receptor binding modalities of FPR1, KISS1R, GRPR and GPR18. In a first step, the Swiss-Model Server [33–35] was used to build homology based receptor models. The target sequences (see Supplementary Data 2) were submitted to the server and the automated mode was used to identify 333, 342, 327 and 279 structural templates for FPR1, KISS1R, GRPR and GPR18, respectively.

**Figure 5:**
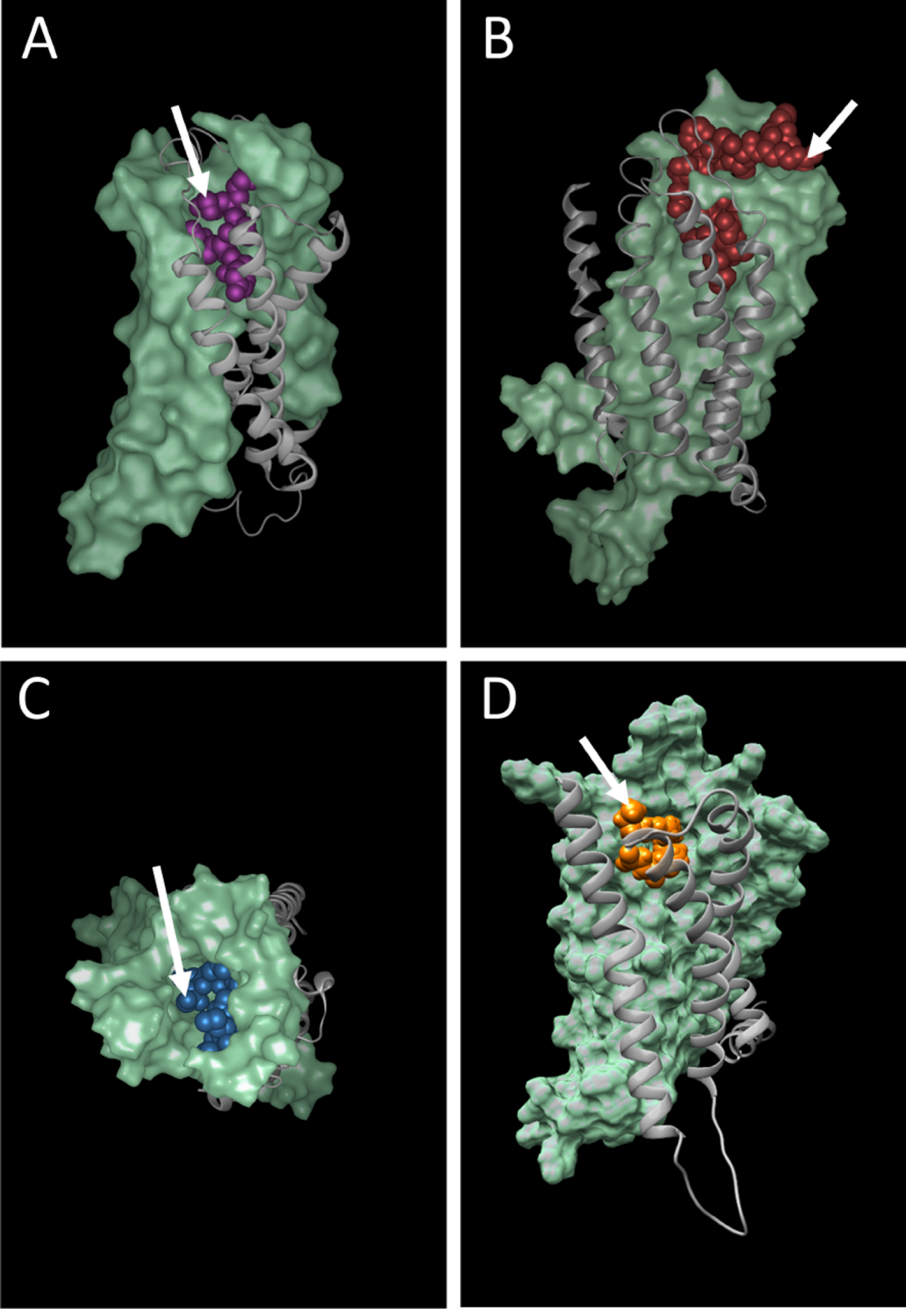
Docking of peptide and lipid ligands into their cognate receptors (**A**) FPR1, (**B**) KISS1R, (**C**) GRPR and (**D**) GPR18. Arrows depict putative anti-cancer agent attachment sites on the N-terminal amino group of uPAR88-92 (A) and neuromedin C (C); the C-terminal carboxy group of kisspeptin14 (B); the C20 atom of arachidonic acid in N-Arachidonylglycine (D).

In a second step, the templates with the highest global quality estimation scores (GMQE) [35] were selected for modelling and the quality of the structures were ranked using the QMEAN scoring function [34]. The highest scoring models were taken forward for docking experiments. The public CABS-dock server [36] was used for docking studies with the peptide ligands uPAR88-92, kisspeptin14 and neuromedin C against the cognate receptors FPR1, KISS1R and GRPR, respectively (Figure 5A–5C). The SwissDock server [37, 38] was used for docking of the endogenous lipid ligand N-Arachidonylglycine (NAGly) [26] against GPR18 (Figure 5D).

Peptide ligands offer the possibility to produce fusion proteins with ELPs or other self-assembling proteins. Proof of concept for this has already been provided in our earlier work, when the N-terminal amino acid of GRP (neuromedin C) was successfully fused to ELP micelles and increased uptake into prostate cancer cells demonstrated [22]. Even though GRP appeared to be buried within the binding site (5C) it was possible to use the N-terminal amino acid for conjugation while maintaining full ligand activity. Potentially, the uPAR88-92 (SRSRY) pentapeptide [39] (Figure 5A) may behave similarly when linked to nanoparticles via the N-terminal amino group, although this hypothesis remains subject to experimental testing. In contrast, the slightly larger kisspeptin14 (5B) seems very well suited for chemical linkage to nanocarriers via the C-terminal amino acid fully exposed on the receptor surface. The model for GPR18 suggests the attachment of a relatively polar linker to the C20 carbon to bridge the linkage to potential binding groups on nanoparticles.

## DISCUSSION

The sheer amount of gene expression data generated using most modern technologies makes it difficult for a single laboratory to exploit all of the information generated. Collection of data from multiple datasets within repositories allows for data retrieval and combination, potentially reaching higher statistical significance with meta studies designed for specific biological questions. We have outlined a process to systematically identify overexpressed GPCRs in cancer tissue.

GPCRs have been recognized as emerging cancer therapeutic target molecules. Several studies show the involvement of GPCR activity in cancer development [32]. They regulate a broad range of cellular signal transduction processes and can be of paramount importance for tumor initiation and progression. Thus, it comes of no surprise that several successful anti-cancer drugs target GPCRs, such as Plerixafor targeting the CXCR4 gene product. However, in contrast to most oncogenes, it is not only gene mutations which drive the GPCR’s tumorigenic capacity. Overexpression of GPCRs or excess ligand production can induce oncogenic transformation. As such, overexpressed GPCRs may not only serve as direct targets for anti-cancer drugs but can be harnessed to guide anti-cancer drugs to cancer cells. Receptor-bound drug carrying ligands will be internalized through endocytosis and the drug released e.g. by the acidic environment within endosomes. However, depending on the drug, internalization may not be necessary. For example, some drug carriers might be immobilized on the cellular surface and deliver the anti-cancer compounds into the acidic tumor environment, or in a radiation based therapy, a drug only needs to reach the surface of a cancer cell by active targeting to perform its action.

Here, we showed the application of a meta study of gene expression data for the identification of overexpressed GPCRs in prostate cancer and breast cancer tumor cells. The rationale that GPCR receptor ligands coupled to an anti-cancer compound or nanocarrier would specifically target and combat cancer cells may pave the way for more effective therapeutics.

The basis for the presented approach lies (1) in the organized collection of data from public sources and (2) in the strength of computer programs to select and order the data according to one’s requirements. As data acquired in various laboratories using different protocols may vary considerably due to many uncontrollable influences inherent of biological samples, we defined quantitative and qualitative threshold levels to account for these variations and to therefore strengthen the significance of the data. For example, only data sets also including control samples measuring gene expression in healthy tissue have been taken into account. Furthermore, signal transduction molecules such as GPCRs are expressed below average (Figures 3B and 4B). Specifically, the mean expression of all genes was calculated to be approximately 5.5 (log2 expression), whilst the average of all GPCRs was lower by about 2 fold (ca. 4.5 log2 expression). Comparison of the maxima revealed an approximate expression difference of 16 fold (log2 of 14 vs 10). Cellular GPCR expression levels must be maintained relatively low for most of the time. In this way, cells can react quickly to agonists for downstream signal transduction. Subtle perturbations in their expression levels may therefore be a significant sign of altered cell state such as occurs when a healthy cell turns into a tumor cell. We reasoned that a 50% increase in expression of GPCRs in cancer cells relative to healthy cells is a sign for this kind of switch of a cellular state. To account for this relatively low cutoff level, only GPCR genes overexpressed in at least 4 patient sample groups were selected for further consideration. We noted that increasing the cutoff level for overexpression to a factor of 2 would lead to approximately the same number of GPCR genes in the final pool although the initial number of overexpressed GPCR genes would have dropped substantially from 186 to 70. *P*-values were calculated to assess differential expression of each GPCR gene when cancer tissue was compared to normal tissue. The *p*-values ranged from < 0.0005 to 0.958 with about 70% of the values being < 0.1. Whilst high values indicate differences of lower significance between the samples, they also reflect the very strong heterogeneity that often occurs between cancer samples. Under these circumstances, large *p*-values will be likely obtained with small sample groups as indicated in Table 1. In fact, the statistical analysis performed here has to be interpreted with regard to such heterogeneity. The selection of the GPCR genes is based on data of a population of cancer samples and serves to narrow down possible candidates for targeted treatment. While some of the selected GPCRs may function as a target in most cancer patients, others may only be overexpressed in 30% or less of all cancer tissues. However, in the context of personalized cancer treatment, the consideration of these less frequently overexpressed receptors are of high practical value. Our findings need to be confirmed through experimental testing. Finally, when anti-cancer drugs are actively targeted to GPCRs on cancer cells, it is of advantage to choose receptors with a general low expression in healthy tissues, as this will minimize possible side effects.

Two groups of overexpressed GPCRs can be expected when following the described selection procedure (Figure 2). (1) GPCRs that have been described to have a functional role in cancer initiation and progression and (2) GPCRs with no known causal connection to the tumorigenic state of a cell. Members of both groups are well suited for the purpose of serving as a cellular lighthouse to guide anti-cancer drug-loaded receptor ligands to the tumor cells.

Based on the present knowledge, we have selected GPCRs from both groups. For example, the Gastrin Releasing Peptide Receptor (GRPR) is overexpressed in various cancer type cells and was suggested to play a significant role in metastasis. Also, the KISS1 receptor was recently shown to promote breast cancer due to overexpression [40]. The selection of these two examples as well as others (Table 4) indicate the validity of our algorithm. On the other hand, an involvement of FPR1, GPR18 and others (Table 4) in cancer promotion has not been described. All of the finally selected GPCR genes have either peptidic or lipidic ligands that can be relatively easily conjugated to drug nanocarriers or directly to anti-cancer small molecule drugs. In fact, it has been recently shown that GRPR on prostate cancer cells can be targeted with hybrid elastin-like polypeptide/liposome nanoparticles via a GRP-ELP fusion protein [23].

Our approach has some limitations. We are aware that these kind of expression studies may miss potentially overexpressed GPCRs whose mRNA expression levels stay below our arbitrary selection criteria of factor 1.5. On the other hand, enhanced mRNA expression may not necessarily lead to enhanced protein levels. Protein measurements are needed to confirm our data before designing targeted therapeutics. The systematic attachment of therapeutic molecules to ligands for overexpressed GPCRs would pave the way to personalized therapeutics in cancer. Patients can easily be tested for overexpression and ligand binding ability of the candidate GPCRs and treated accordingly. Whether or not the GPCR systems used for this kind of therapeutic approach may also be used as a cancer diagnostic or even cancer prognostic tool is another interesting aspect to address in future work.

## METHODS

### Data set search strategy

The NCBI GEO database was systematically searched to identify Entrez GEO DataSets with micro array expression data relevant for prostate and breast cancer. The search was focused on the GPL570 platform (single channel array) which represented >90% of all non-olfactory GPCR genes and with >120,000 available samples provided sufficient data. Only studies with information from primary cancer tissue were considered in our analysis. Hence, experiments with *in vivo* cell lines and xenograft models were excluded. Furthermore, studies without control samples (e.g. healthy tissue) or studies with <10 samples were not considered. Finally 6 data sets were selected for prostate cancer and 5 for breast cancer (Table 1).

### Search for overexpressed GPCR genes

The CEL files of all datasets listed in Table 1 were retrieved from the NCBI GEO archive. The ArrayStar software (DNAStar Inc, Madison, WI, USA) was used to normalize the data with the robust multi-array average (RMA) method [24] to assure consistent handling of all data sets. Affymetrix annotation files were used to retrieve gene specific expression data with official gene symbols. The 7tm and GPCR Protein Family (Pfam) domains were used to identify all GPCRs listed in the Ensemble database (ensemble.org) and 755 GPCR genes were compiled including orphan, taste, olfactory and vomeronasal receptors. A subset of 437 receptors were represented by at least 1 DNA probe on the GPL570 platform.

### Statistical analysis

Patient samples from different studies were stratified into groups of distinct subtypes and fold expression was calculated between cancer tissues and the corresponding normal tissues. An unpaired, two-tailed, equal variance Student’s *t*-Test was applied to assess the significance of differentially expressed GPCR genes.

### Homology modeling and docking

Receptor protein sequences were submitted to the Swiss-Model Server [33–35] and suitable templates were automatically searched in the SWISS-MODEL template library (SMTL, version 2017-10-23, last included PDB release 2017-10-13) using the Blast [41] and HHBlits [42] methods in parallel. The templates with the highest quality according to the global quality estimation score (GMQE) have then been selected for model building. Models were built based on the target-template alignment using ProMod3 and the global and per-residue model qualities were assessed using the QMEAN scoring function [34]. Finally, the CABS-dock server [36] was used for docking studies with selected peptide ligands against receptor models. The SwissDock server [37, 38] was used for docking of lipid ligands. Various docking results were clustered according to the binding site and modality on the receptor. Visual checks of the structures were performed to discard clusters which showed binding in unexpected sites. Top ranked dockings from the finally prioritized clusters were rendered with Protean 3D (DNAStar Inc.) or Chimera (UCSF), and the structures were used to assess potential chemical conjugation for receptor targeting.

## SUPPLEMENTARY MATERIALS AND FIGURES



## Abbreviations

BrCa: breast cancer
ELP: elastin-like polypeptide
EPR: enhanced permeability and retention effect
GEO: gene expression omnibus
GMQE: global quality estimation scores
GPCR: G-protein-coupled receptor
GRP: gastrin-releasing peptide
GRPR: gastrin-releasing peptide receptor
HPA: Human Protein Atlas
IDC: invasive ductal carcinoma
NAGly: N-arachidonylglycine
PCa: prostate cancer
RMA: robust multi-array
