# Supplementary material for: Large set data mining reveals overexpressed GPCRs in prostate and breast cancer: potential for active targeting with engineered anti-cancer nanomedicines
Source: Oncotarget. 2018 May 18;9(38):24882–97. doi: 10.18632/oncotarget.25427 (PMC5982759; doi:10.18632/oncotarget.25427)
Supplement: Supplementary file 1 [file oncotarget-09-24882-s001.pdf]

Large set data mining reveals overexpressed GPCRs in prostate and breast cancer: potential for active targeting with engineered anti-cancer nanomedicines

SUPPLEMENTARY MATERIALS

Supplementary Data

Supplementary Data 1: Protein expression data from the Human Protein Atlas (HPA), Healthy tissue

	Peptide Receptors																																						Lipid receptors										Control	
	PCa										BrCa																		P&BrCa										BrCa											
	AGTR1		C3AR1		FPR1		F2R		SSTR1		CCR1		CCR2		CCR5		CCR7		CXCR3		GPR171		GPR19		GPR37		KISS1R		CXCR4		F2RL1		F2RL2		FPR3		GRPR		GPR18		GPR84		LPAR2		LPAR5		S1PR3		GAPDH	
	P	R	P	R	P	R	P	R	P	R	P	R	P	R	P	R	P	R	P	R	P	R	P	R	P	R	P	R	P	R	P	R	P	R	P	R	P	R	P	R	P	R	P	R	P	R	P	R	P	R[1)]
Cerebral cortex	1	0	0	14	0	14	1	13	2	14		10	1	1		2	0	0	0	0	1	1		9	2	79		1		11	1	3		0		4		0	0	0	1	1	3	5	2	8		7	2	25
Hippocampus	0		0		0		0		0				1				0		0		0				0						1								0		1		2		2				2	
Caudate	0		0		0		0		0				1				0		0		0				0						1								0		2		2		1				2	
Cerebellum	0		0		0		0		0				1				0		0		0				1						1								0		2		1		0				2	
Thyroid gland	0	12	1	5	0	4	0	25	0	0		2	0	2		1	0	2	0	1	0	1		0	1	0		0		27	1	14		0		4		0	0	0	0	0	2	11	2	2		6	2	12
Parathyroid gland	0	5	0	8	0	2	0	7	0	0		3	0	0		1	0	0	0	0	0	0		0	0	0		0		9	1	6		0		3		0	0	0	1	0	2	20	2	1		2	2	20
Adrenal gland	0	50	0	22	0	9	0	17	1	0		6	0	4		3	0	0	0	1	0	2		1	0	0		0		236	1	2		1		16		0	0	0	1	1	2	3	2	3		34	2	13
Appendix	0	3	2	88	1	182	0	16	2	0		58	0	53		21	1	92	0	8	0	31		3	0	0		0		407	2	8		3		34		0	0	17	2	12	2	26	2	7		15	2	17
Spleen	0	13	0	23	1	75	2	94	0	1		41	2	21		13	3	40	2	9	0	19		1	0	5		0		293	1	5		0		23		0	2	15	0	1	2	28	2	8		19	2	8
Lymph node	0	1	2	21	1	11	2	17	0	0		12	2	19		16	3	98	2	18	0	22		2	0	0		0		433	1	2		0		15		0	2	52	0	0	2	20	2	10		14		17
Bone marrow	0	0	1	22	2	95	2	2	0	0		14	2	2		1	1	9	2	4	0	1		1	0	0		0		773	1	1		0		0		0	0	7	3	15	2	34	2	1		2	2	29
Tonsil	0	0	1	8	1	7	1	8	1	0		9	3	10		9	3	80	2	5	1	12		2	0	0		0		515	1	2		0		11		0	2	33	0	1	2	14	3	11		9	2	17
Heart muscle	0	6	1	4	1	2	0	19	0	1		2	1	1		1	0	1	0	0	0	0		0	0	0		0		11	1	2		1		3		0	0	0	1	0	2	1	1	1		7	2	30
Skeletal muscle	0	3	1	0	1	0	0	1	0	0		0	0	0		0	0	0	1	0	0	0		0	0	0		0		1	1	0		0		0		0	0	0	1	0	2	0	2	0		1	2	99
Smooth muscle	0	7	0	34	0	43	0	27	0	1		21	0	15		5	0	2	0	1	2	2		0	1	3		0		40	2	4		6		13		1	0	0	0	3	1	3	1	2		52	2	19
Nasopharynx	0		1		0		2		1				1				0		0		0				0						1								0		0		2		2				2	
Bronchus	0		1		0		0		1				1				0		1		0				0						1								0		0		2		2				2	
Lung	0	10	1	39	1	32	0	30	0	8		27	1	13		11	3	4	1	5	0	7		0	0	1		0		48	1	6		1		41		0	0	1	0	1	2	11	2	3		9	2	8
Liver	0	51	0	9	0	8	0	12	0	3		9	0	1		2	0	1	0	0	0	2		0	1	4		0		26	1	4		1		11		0	0	1	0	0	0	1	0	1		3	2	7
Gallbladder	0	10	0	41	0	35	0	62	1	1		18	1	8		8	0	8	0	2	0	11		1	0	1		0		133	2	47		11		30		0	0	2	0	2	3	20	2	3		23	2	18
Pancreas	0	1	1	2	0	2	0	2	2	1		1	0	0		0	0	0	0	0	0	0		0	0	0		0		2	1	5		0		1		4	0	0	0	0	2	4	2	0		3	3	1
Esophagus	0	3	0	7	0	8	0	12	1	0		4	1	2		2	0	2	0	1	2	3		1	1	7		0		38	1	8		1		6		0	0	1	0	0	0	8	3	8		21	2	32
Oral mucosa	0		0		0		0		0				1				0		0		1				0						0								0		0		0		2				1	
Salivary gland	0	1	0	1	0	1	0	3	1	0		1	1	2		1	0	0	0	1	0	1		0	0	0		0		14	1	3		0		1		0	0	1	0	0	1	4	1	2		2	2	5
Rectum	0	5	1	12	1	3	0	13	2	3		8	0	8		5	0	6	0	1	0	6		1	0	1		0		55	2	59		2		20		1	0	1	1	1	2	9	3	9		4	2	17
Stomach	0	1	1	7	1	2	0	9	3	21		4	1	7		6	0	5	0	3	0	6		0	0	0		0		54	3	42		1		7		1	0	2	0	0	3	33	2	7		4	2	6
Duodenum	0	1	1	9	0	2	0	14	3	15		7	0	11		4	0	1	0	5	0	6		0	0	0		0		13	3	46		2		12		0	0	1	0	0	2	10	3	10		5	2	16
Small intestine	0	2	1	15	1	4	0	20	3	9		7	0	9		7	0	3	0	7	0	15		0	0	1		0		56	2	45		3		17		0	0	2	0	0	2	12	3	9		4	2	16
Colon	0	4	1	10	1	4	1	13	2	4		5	0	7		3	0	4	0	2	0	5		1	0	1		0		32	2	60		1		13		0	0	1	0	0	1	7	3	9		4	1	15
Kidney	0	21	0	5	0	3	2	20	1	2		5	1	2		1	0	0	0	0	0	1		0	0	0		0		16	2	34		0		4		0	0	0	0	0	2	2	2	1		14	2	13
Urinary bladder	0	10	0	27	0	23	0	23	2	1		14	1	13		7	0	13	0	2	0	9		0	0	0		0		101	1	12		2		21		0	0	4	1	2	2	10	2	3		16	2	14
Epididymis	0	4	0	3	1	1	1	5	2	1		2	0	2		1	0	1	0	0	0	1		0	0	1		0		7	1	6		0		2		3	0	0	0	0	2	3	2	3		8	3	9
Seminal vesicle	0	6	0	9	0	2	1	16	2	1		3	0	3		1	0	1	0	0	0	1		0	0	1		0		16	1	16		0		4		0	0	0	1	0	1	6	2	2		17	2	16
Testis	0	4	1	6	1	4	2	16	2	0		3	0	3		2	0	4	0	1	0	1		8	1	28		0		6	1	1		0		5		0	0	25	0	1	2	41	2	1		5	3	9
Prostate	0	9	0	6	0	3	0	12	2	1		3	1	3		3	0	2	0	1	0	3		0	1	1		0		51	1	8		0		4		1	0	1	0	1	3	15	2	1		23	2	13
Fallopian tube	0	4	0	10	0	7	0	6	1	1		4	0	3		3	0	2	0	1	0	2		0	0	2		0		100	1	13		1		3		0	0	0	0	0	2	11	2	3		17	2	15
Cervix, uterine	0	6	0	18	0	8	0	24	1	1		4	2	4		3	0	2	0	1	1	8		0	0	1		0		55	1	5		2		9		1	0	0	0	0	2	8	2	2		34	2	12
Breast	0	17	1	13	0	5	0	22	1	0		5	1	5		3	0	2	0	1	0	2		0	0	1		0		47	0	10		5		7		3	0	0	0	0	2	6	2	2		15	2	9
Vagina	0		0		0		0		1				1				0		0		2				0						0								0		0		0		0					
Endometrium	0	4	1	10	0	3	2	38	1	2		4	1	3		2	0	2	0	1	0	2		0	0	2		0		58	1	5		4		9		1	0	1	0	0	2	6	2	1		26	2	9
Ovary	0	5	0	6	0	1	0	4	1	1		3	0	2		2	0	1	0	1	0	1		0	0	0		0		16	1	0		0		2		0	0	0	0	0	1	7	1	1		2		8
Placenta	0	112	1	49	0	44	2	15	0	4		37	1	2		1	0	2	0	0	2	1		0	1	13		0		80	1	5		0		8		0	0	0	0	4	2	6	2	8		22	2	16
Adipose tissue	3	42	0	35	0	62		13	0	1		25	0	5		2	0	4		1	0	3		0	0	1		0		37	0	1		0		13		0	0	1	1	6	1	5	1	1		23	2	8
Soft tissue	3		1		0		1		1				0				0		0		0				0						0								0		1		1		2				2	
Skin	0	3	0	1	1	1	2	53	1	0		1	1	3		1	0	3	0	1	0	2		1	0	4		0		4	0	40		3		3		0	0	1	1	1	1	16	1	7		2	2	12

White boxes: No protein or RNA expression data available from HPA. P: Protein expression levels from 0 (no expression detected) to 3 (high expression level). R: RNA expression levels (tags per million). 1) Data divided by factor 100.

Supplementary Data 2: Protein sequences for 3D modeling

FPR1

METNSSLPTNISGGTPAVSAGYLFLDIITYLVFAVTFVLGVLGNGLVIWVAGFRMTHTVTTISYLNLAVADFCFT
STLPFFMVRKAMGGHWPFGWFLCKFVFTIVDINLFGSVFLIALIALDRCVCVLHPVWTQNHRTVSLAKKVIIGPWV
MALLLTLPVIIRVTTVPGKTGTVACTFNFSPWTNDPKERINVAVAMLTVRGIIRFIIGFSAPMSIVAVSYGLIATKIHKQG
LIKSSRPLRVLSFVAAAFFLCWSPYQVVALIATVRIRELLQGMYKEIGIAVDVTSALAFFNSCLNPMLYVFMGQDFRE
RLIHALPASLERALTEDSTQTSDTATNSTLPSAEVELQAK.

GRPR

MALNDCFLLNLEVDHFMHCNISSHSADLPVNDDWSHPGILYVIPAVYGVIILIGLIGNITLIKIFCTVKSMRNVP
NLFISSLALGDLLLLITCAPVDASRYLADRWLFGRIGCKLIPFIQLTSVGVSVFTLTALSADRYKAIVRPMDIQASHAL
MKICLKAAFIWIISMLLAIPEAVFSDLHPFHEESTNQTFISCAPYPHSNELHPKIHSMASFLVFYVIPLSIISVYYYFIAK
NLIQSAYNLPVEGNIHVKKQIESRKRLAKTVLVFVGLFAFCWLPNHVIYLYRSYHYSEVDTSMLHFVTSICARLLAF
TNSCVNPFALYLLSKSFRKQFNTQLLCCQPGLIIRSHSTGRSTTCMTSLKSTNPSVATFSLINGNICHERYV.

KISS1R

MHTVATSGPNASWGAPANASGCPGCGANASDGPVPSPRAVDAWLVPLFFAALMLLGLVGNSLVIYVICRHKP
MRTVTNFYIANLAATDVTFLLCCVPFTALLYPLPGWVLGDFMCKFVNYIQQVSVQATCATLTAMSVDRWYVTVFPL
RALHRRTPRLALAVSLSIWVGSAAVSAPVLALHRLSPGPRAYCSEAFPSRALERAFALYNLLALYLLPLLATCACYAA
MLRHLGRVAVRPAPADSALQGQVLAERAGAVRAKVSRLVAAVVLLFAACWGPIQLFLVLQALGPAGSWHPRSYAAY
ALKTWAHCMSYSNSALNPLLYAFLGSHFRQAFRRVCPCAPRRPRRPRRPGPSDPAAPHAELLRLGSHPAPARAQKPG
SSGLAARGLCVLGEDNAPL.

GPR18

MITLNNQDQPVPFNSSHPDEYKIAALVFYSCIFIIGLFVNITALWVFSCTTKKRTTVTIYMMNVALVDLIFIMTLP
FRMFYYAKDEWPFGEYFCQILGALTVFYPSIALWLLAFISADRYMAIVQPKYAKELKNTCKAVLACVGVWIMTLTT
TTPLLLLYKDPDKDSTPATCLKISDIIYLKAVNVLNLTRLTFFFLIPLFIMIGCYLVIIHNLLHGRTSKLKPKVKEKSIRIII
TLLVQVLVCFMPFHICFAFLMLGTGENSYNPWGAFTTFLMNLSTCLDVILYYIVSKQFQARVISVMLYRNYLRSMR
RKSFRSGSLRSLSNINSEML.
